# The early development of executive function and its relation to social interaction: a brief review

**DOI:** 10.3389/fpsyg.2014.00388

**Published:** 2014-04-29

**Authors:** Yusuke Moriguchi

**Affiliations:** ^1^Department of School Education, Joetsu University of EducationJoetsu, Japan; ^2^Precursory Research for Embryonic Science and Technology, Japan Science and Technology AgencyTokyo, Japan

**Keywords:** executive function, social interaction, preschool children, theory of mind, cognitive development

## Abstract

Executive function (EF) refers to the ability to execute appropriate actions and to inhibit inappropriate actions for the attainment of a specific goal. Research has shown that this ability develops rapidly during the preschool years. Recently, it has been proposed that research on EF should consider the importance of social interaction. In this article, recent evidence regarding the early development of EF and its relation to social interaction has been reviewed. Research consistently showed that social interaction can influence EF skills in young children. However, the development of EF may facilitate the cognitive skills that are important for social interaction. Taken together, there might be functional dependency between the development of EF and social interaction.

## INTRODUCTION

Executive function (EF) is a complex cognitive control responsible for making adaptive changes in physical and social environments. It enables us to execute appropriate actions, and to inhibit inappropriate actions, to attain a goal (Dempster, 1992). Extensive evidence suggests that EF develops rapidly in the preschool years, with adult-level performance being achieved during adolescence (Anderson, 2002; Zelazo et al., 2003). The development of EF is supported by the maturation of the prefrontal cortex in preschool children as well as school-aged children (Diamond, 2002; Durston et al., 2006; Moriguchi and Hiraki, 2009).

One important issue in EF research is its structure. Adult research has shown that EF is not unitary. It consists of some sub-components, such as inhibition, shifting, and updating (working memory; Miyake et al., 2000; Miyake and Friedman, 2012). Although their studies focused on healthy adult populations, studies for elderly people and school-aged children also confirmed the Miyake et al. (2000) model (Lehto et al., 2003; Huizinga et al., 2006). However, in preschool children, a single-factor model (general EF) may be more appropriate (Wiebe et al., 2008). Alternatively, in younger children, the model of “conflict” EF and “delay” EF may be useful. The former refers to inhibiting a prepotent response while activating conflicting novel responses. The conflict EF is indexed by Stroop-like tasks or rule switching tasks. The latter refers to simply inhibiting responding, which is indexed by a delay of gratification (Carlson and Moses, 2001).

In the previous studies, problem-solving tasks, such as the Dimensional Change Card Sort (DCCS) task or the Day–Night Stroop task (Gerstadt et al., 1994; Zelazo et al., 1996; Kirkham et al., 2003) were extensively used. In the DCCS task, children are asked to sort cards that have two dimensions such as color and shape (e.g., red boats, blue rabbits). There are two phases in the task. In the first phase, children are asked to sort cards according to one dimension (e.g., color), for five or six trials. In the second phase, they are asked to sort the cards according to the other dimension (e.g., shape), for five or six trials. Research has shown that 3-year-olds tend to perseverate on the first dimension whereas older children do not show such perseveration. The researchers argued how and why young children made perseverative errors in the DCCS (Kirkham et al., 2003; Zelazo et al., 2003; Kloo and Perner, 2005).

Moreover, recently, the neural basis of EF in young children has been extensively examined (Moriguchi and Hiraki, 2011, 2014; Espinet et al., 2012; Buss et al., 2014). Indeed, research using near-infrared spectroscopy has shown that the performances of the DCCS tasks were significantly correlated with activations in the lateral prefrontal areas (Moriguchi and Hiraki, 2009). Further, the amplitude of N2 components measured by event-related potentials differed between children who passed and failed the DCCS tasks (Espinet et al., 2012). Previous research consistently showed that activations in the prefrontal cortex are important for successful performances in EF tasks.

## SOCIAL INTERACTION INFLUENCES EF IN LATER DEVELOPMENT

However, until recently, how EF develops in social interaction has been largely neglected. This is in spite of the fact that several theorists proposed that humans are in nature social and develop through social interaction (Vygotsky, 1978; Tomasello, 2009). It has been proposed that higher mental functions, such as self-regulation, develop within the context of interpersonal activity (Vygotsky, 1978). In this view, interpersonal interaction may facilitate internalizing some views of another person’s perspective on reality, which improves the development of higher mental functions. This process is clearly manifested in a parent-child relationship. Parents provide children with alternative perspectives regarding how to deal with a given problem that can be internalized by a child. When children define an inappropriate goal, parents may direct children’s attention to an appropriate goal. The external dialog between parent and child may go on to be internalized by the child in later development.

Lewis and Carpendale (2009) proposed that research on EF should consider the roles of social interaction. Indeed, there is evidence that social interaction between parents and children influences the development of EF. According to Roskam et al. (2014), there might be two possible dimensions of how interaction between parents and children influences EF in later development: supportive parenting and negative control. The former includes scaffolding, acceptance, and autonomy, which may facilitate children’s development of EF. For example, Landry et al. (2002) showed that maternal verbal scaffolding affected EF skills such as search retrieval, mediated by children’s verbal and non-verbal problem-solving skills. Bernier et al. (2010) examined whether maternal sensitivity, mind-mindedness and scaffolding at 12 to 15 months of age predicted children’s EF skills, such as working memory and set shifting. Sensitivity refers to the tendency to read the child’s needs and respond sensitively and appropriately (Ainsworth et al., 1978). Mind-mindedness refers to maternal appropriate use of mental language to their infants (Meins et al., 2003). The results revealed that scaffolding was the strongest predictor of the development of EF. Moreover, Hughes and Ensor (2009) reported that maternal scaffolding as well as other factors, such as imitative learning, plays an important role in the development of EF.

The other dimension about the relationship between parenting and EF in later development may be negative control. Negative control refers to parenting through coercion and punishment. It has been repeatedly shown that such parenting may lead to children’s negative behaviors in later development (Gershoff, 2002). In terms of EF, Roskam et al. (2014) reported that negative control parenting may have negative impact on children’s EF skills in later development. Specifically, the longitudinal research has shown that changes in negative parenting (e.g., frequent use of punishment) induced negative changes in inhibitory skills. Similarly, Blair et al. (2011) reported that positive (e.g., sensitivity) and negative (e.g., intrusiveness) parenting during infancy influenced EF and IQ in later development. The effect of negative parenting may be due to that such parenting may fail to provide children with the opportunity to control their actions.

Despite insufficient evidence, the previous studies suggest that social interaction, specifically interaction with parents, influenced the development EF skills in young children. Although the previous studies have examined the effect of parenting on children’s EF, interaction with peers can be also important. Indeed, some researchers proposed that collaborative learning can enhance cognitive development, such as traditional Piagetian tasks (Doise and Mugny, 1984). In collaborative learning, each individual have different opinion in a given tasks, which induces social conflict. However, such situations may provide children with the impetus to have different perspectives, which may lead to improvement of the performances. According to Qu (2011), there are several reasons why collaboration with another person can facilitate children’s executive control. For example, children may be aware a goal of the task and can have another person’s perspectives thorough collaboration with another person, which lead to more efficient executive control. Future research should focus on the role of peer interaction in the development of EF.

## SOCIAL LEARNING AND EF

The research above showed that parenting may play an important role in EF skills in later development. Such previous research clarified the long-term relationship between EF and social interaction, but it is still unclear how social interaction directly influenced children’s behaviors that require executive control. Thus, I introduce the experimental evidence in the context of social and imitative learning. It is well known that children “overimitate” another person’s behaviors (Horner and Whiten, 2005; Lyons et al., 2007; McGuigan et al., 2007). Overimitation refers to children’s tendency to reproduce an adult’s obviously irrelevant actions. For example, in a tool-use task, chimpanzees and 3- to 4-year-old children were asked to observe an experimenter using a tool to obtain a reward from a complex-structured box (Horner and Whiten, 2005). In the demonstration, some actions were causally relevant to obtain a reward, and other actions were causally irrelevant. When they performed the actions, chimpanzees only reproduced relevant actions whereas human children reproduced both relevant and irrelevant actions. Research on overimitation suggests that social and imitative learning may be so powerful that children may fail to control their behaviors after such learning.

Moriguchi et al. (2007) examined whether children’s executive control might be influenced by learning from another person’s actions. They modified the DCCS. In the modified social DCCS task, during the first phases, instead of sorting the cards by themselves, preschoolers watched an adult model sorting the cards according to one dimension (e.g., shape). During the second phases, children were asked to sort according to a different dimension (e.g., color). The results showed that most 3-year-olds perseverated sorting according to the observed dimension, as in the standard DCCS task. Thus, 3-year-old children used the observed rules even though they were asked to use the different rules. On the other hand, more than half of the 4-year-old children and most of the 5-year-old children did not use the observed rules, and sorted the cards according to the instructed, second rules.

Interestingly, children’s behaviors were significantly affected by a model’s mental states (Moriguchi et al., 2007). For example, children were more likely to use the observed rules when they observed a model who was confident with the rule she used than when they observed a model who lacked confidence with the rules. Moreover, the performance on the modified DCCS tasks is significantly correlated with performance on the standard DCCS tasks (Moriguchi and Itakura, 2008).

Children’s executive control process could be affected by a human’s actions, but not a robot’s actions. Moriguchi et al. (2010) showed that children who observed a robot sorting according to one dimension had no difficulty in sorting the cards according to a different dimension. Moriguchi et al. (2010) reported that the effects of demonstration by an android (a robot with human appearance) were stronger than those by a robot, but weaker than those by a human model. The authors explain the results in terms of a sociocognitive perspective that children perseverate on the human model’s rule because they mentally simulate the model’s actions while watching. On the other hand, the children’s actions were not affected by the robot’s actions because the robot did not induce young children’s simulative processes.

There might be some cultural differences in performance on the social DCCS. Moriguchi et al. (2012) gave 3- and 4-year-olds in Canada and Japan the standard version of the DCCS and the social version of the DCCS. Results indicated that Canadian children displayed the perseverative behaviors in the social DCCS, but their effects were relatively weaker than those of Japanese children. On the other hand, performance on the standard DCCS was similar between the two countries. Although the general developmental trajectory may be common in two cultures, the results can be interpreted in terms of cultural psychology theories. People in Western cultures tend to have a more “independent” view of the self, whereas people in Asian cultures are likely to have a more “interdependent” view (Markus and Kitayama, 1991). In interdependent cultures, people may recognize that their behaviors can be affected by others’ behaviors. On the other hand, in independent cultures, people may tend to believe that their behaviors are independent from others’. Thus, Canadian children may be more likely to separate themselves from another person than Japanese children.

The effects of social interaction on EF were reported using a Stroop-like Black/White task (Moriguchi, 2012). In this task, children were asked to respond to a pair of pictures: in the black/white task, for example, children had to respond “black” when shown a white card, and respond “white” when shown a black card. Children were told to suppress the tendency to respond according to what color the card was and instead activate a conflicting response (Simpson and Riggs, 2005). This study compared the standard condition to an interference condition. In the interference condition, children observed incorrect demonstrations, where the demonstrator responded with “black” to a black card, and “white” to a white card; they were then given the black/white task. The results revealed that children in the interference condition performed worse than those in the neutral condition.

Research suggests that interaction with a human, but not with non-human agents, can affect children’s EF skills. In addition, culture may influence the relationship between EF and social interaction. Taken together with the evidence of parenting, social interaction may have a strong impact on children’s executive control.

## EF INFLUENCES SOCIAL INTERACTION

The research reported above suggests that social interaction can affect children’s EF skills. Conversely, there is accumulating evidence that EF skills may facilitate the development of social interaction. The most well-known case is that EF was significantly correlated with theory of mind (ToM; Frye et al., 1995; Hughes, 1998; Carlson and Moses, 2001; Sabbagh et al., 2006; Benson et al., 2012). ToM refers to the ability of children to be aware that they or other individuals can have mental states, such as false beliefs. Extensive research indicates that representative false belief understanding improves markedly during the preschool years (Wellman et al., 2001).

Given the existing evidence regarding the relationship between EF and ToM, theorists argued how EF was related to ToM. First, the development of false belief understanding may contribute to improvement in children’s EF (Perner et al., 2002). According to the view, metarepresentational understanding underlying ToM provides the foundation for the development of EF skills. Indeed, Kloo and Perner (2003) reported that training children on ToM tasks leads to improvement in their performance on the DCCS task. Nevertheless, DCCS training also improves children’s performance on ToM tasks. Moreover, longitudinal research has shown that early EF skills (i.e., 2 years of age) predict later ToM abilities (i.e., at 3 years of age) rather than the reverse (Carlson et al., 2004). This evidence may challenge the view that ToM improves EF skills.

Many researchers have speculated that conflict EF is fundamental for the development of false belief understanding. There are mainly two explanations in this view. One explanation is the expression view, by which children fail to perform false belief tasks because children did not have EF skills to deal with the task demands in the false belief tasks. In this view, children do have an understanding of another person’s false belief, but appeared to lack understanding due to their poor executive skills. The other explanation is the emergence view. On this view, EF may be necessary for the emergence of children’s false belief understanding. The recent evidence favors the latter view (Benson et al., 2012; Devine and Hughes, 2014). For example, children’s EF skills are correlated with performance on ToM tasks with fewer executive demands (Henning et al., 2011).

In relation to this point, EF in young children might be correlated with their lying behaviors. Lying involves a false statement with the intention to deceive the recipient while considering the recipient’s psychological state (e.g., knowledge). Talwar and Lee (2008) administered a peeking task, EF tasks, and ToM tasks to 3- to 8-year-old children, and examined the relationship between them. In the peeking task, after the children and an experimenter played a game, the experimenter left the room. The children were told not to peek at a toy while the experimenter was out of the room. After the experimenter returned to the room, children were asked whether they had peeked at the toy. There were two questions. The first question was whether they had peeked at the toy, and the second question was what the toy was. The assumption on the second questions was that if children had not peeked at it, they would not know what the toy was. The results revealed that children’s lying in respond to the first question about peeking was significantly correlated with their EF skills measured by the Day–Night Stroop task and the scores of false belief tasks. The lying in response to the second question was also correlated with the scores of the Day–Night Stroop task. That is, children who developed more EF skills tended to lie more.

In terms of the relationship between EF and communicative behaviors, Moriguchi et al. (2008) examined the correlation between the performance on the DCCS tasks and a yes bias in preschool children. The yes bias is children’s tendency to answer “yes” when they are posed yes/no questions. The bias occurs in spite of knowing that the correct answer is “no” (Fritzley and Lee, 2003; Okanda and Itakura, 2007). Okanda and Itakura (2007) suggested that affirmation including a yes response could be a dominant response and children would not able to inhibit the response. Thus, it was possible that having inhibitory skills help a child to avoid saying the first thing that comes to his or her mind when asked a question by an interviewer. Moriguchi et al. (2008) found that better inhibitory control ability was significantly related to a weaker yes bias even after controlling for age and verbal ability.

Other research showed that EF might be correlated with the development of moral behaviors. Kochanska et al. (1996, 1997) examined the relationship between effortful control (more temperamental aspects of EF) and moral-related behaviors in young children. For example, children’s effortful control assessed by tasks such as delay of gratification were significantly correlated with children’s internalizations of mothers’ prohibitions of refraining from attractive activities (Kochanska et al., 1996). In addition, Kochanska et al. (1997) reported that children’s effortful control at toddler and preschool age longitudinally contributed to conscience development at an early school age. The conscience development was measured by sustaining mundane activities and suppressing desired behaviors. Moreover, children’s effortful control was significantly negatively correlated with antisocial responses on hypothetical moral dilemma tests. Further, children’s views of themselves on moral dimensions were significantly correlated with effortful control.

Taken together, the previous research showed that EF skills are significantly correlated with false belief understanding, lying behaviors, responses in questioning, and the internalization of rules in young children. The causal relationship between these variables is still unclear. It is possible that the development of EF contributes to socio-communicative behaviors, or vice versa, and therefore future research should address the causal relationship. Nevertheless, the previous results suggest that the development of EF is closely related to the development of cognitive skills important for social interaction.

## CONCLUSION AND FUTURE DIRECTION

In sum, previous studies showed that social interaction may facilitate the development of EF. Specifically, maternal scaffolding may be a strong predictor of the development of EF skills. In addition, interaction with a person, not a non-human agent, can be important for children’s EF skills. Conversely, children’s EF skills may also facilitate the development of social interaction skills, such as ToM, communicative behaviors, and moral skills. Taken together, the accumulated evidence suggests functional dependency between the development of EF skills and social interaction.

Future research should utilize social interaction to intervene with children who have lower EF skills. Recently, it has been repeatedly shown that self-control abilities, including EF skills, in young children predict school success and socioeconomic status during adulthood (Blair and Razza, 2007; Moffitt et al., 2011). Thus, several training programs have been proposed to facilitate children’s EF skills (Lillard and Else-Quest, 2006; Thorell et al., 2009; Diamond and Lee, 2011). Some trainings use computer-based programs, and others used school curricula. Given the present review, we suggest that intervention programs that include social interaction can be more useful than those that do not.

The other possible direction for future research is to examine the neural basis of EF skills and its relation to social interaction. The development of EF skills is related to the activations in the prefrontal cortex (Espinet et al., 2012; Moriguchi and Hiraki, 2013). However, it is still unclear which factors affect the development of the prefrontal activations. Given the evidence reported here, maternal scaffolding can affect the development of the prefrontal activations in young children. Moreover, there is an argument regarding whether EF skills share brain regions with social interaction skills, such as ToM (Perner and Lang, 2000). There may be some commonalities in the brain regions, although core brain regions in EF may be different from those in ToM (Miller and Cohen, 2001; Saxe et al., 2006; Kalbe et al., 2010). The previous research was mostly based on adult brain imaging research or neuropsychological evidence, and there was little neuroimaging research in young children. Given that some of the brain regions may begin with broad functionality, and then be specialized to a given stimuli and task (Johnson, 2011), it is possible that the neural basis of EF shares the neural basis of ToM in young children. Thus, future studies should address these possibilities.

## Conflict of Interest Statement

The author declares that the research was conducted in the absence of any commercial or financial relationships that could be construed as a potential conflict of interest.
